# Personality traits associate with behavioral problems in pet dogs

**DOI:** 10.1038/s41398-022-01841-0

**Published:** 2022-02-23

**Authors:** Milla Salonen, Salla Mikkola, Emma Hakanen, Sini Sulkama, Jenni Puurunen, Hannes Lohi

**Affiliations:** 1 Department of Veterinary Biosciences, University of Helsinki, Helsinki, Finland; 2 Department of Medical and Clinical Genetics, University of Helsinki, Helsinki, Finland; 3 Folkhälsan Research Center, Helsinki, Finland

**Keywords:** Psychology, Psychiatric disorders

## Abstract

Personality traits, especially neuroticism, strongly predict psychopathology. The domestic dog (*Canis lupus familiaris* Linnaeus, 1758) is used as a natural model for psychiatric disorders, but the similarity between dog and human personality and the association between dog personality and unwanted behavioral traits, such as fearfulness, aggressiveness, and impulsivity/inattention, remain unknown. This study utilized structural equation modeling (SEM) with survey data of 11,360 dogs to examine the associations and correlations between seven personality and ten unwanted behavioral traits. Personality traits included insecurity, energy, training focus, aggressiveness/dominance, human sociability, dog sociability, and perseverance. Unwanted behavioral traits included fearfulness, noise sensitivity, fear of surfaces/heights, separation anxiety, barking, stranger-directed aggression, owner-directed aggression, dog-directed aggression, hyperactivity/impulsivity, and inattention. We first fitted confirmatory factor models for the unwanted behavioral traits and the best model grouped unwanted behaviors into four latent traits: fear-related behavior, fear-aggression, aggression, and impulsivity/inattention and used this structure in the subsequent SEM model. Especially, insecurity, which resembles the human neuroticism trait, was strongly associated with unwanted behavior, paralleling the association between neuroticism and psychopathology. Similarly, training focus, resembling conscientiousness, was negatively related to impulsivity/inattention, and aggressiveness/dominance was associated with aggressive behaviors, resembling associations of conscientiousness and agreeableness with attention deficit hyperactivity disorder and aggression-related psychopathology, respectively. These results indicate that dog personality traits resemble human personality traits, suggesting that their neurological and genetic basis may also be similar and making the dog a suitable animal model for human behavior and psychiatric disorders.

## Introduction

Although personality psychologists do not completely agree on the structure of human personality, the five-factor model of personality has gained the most support [1]. This five-factor model is a hierarchical model that includes two metatraits (stability and plasticity), five traits (neuroticism, agreeableness, conscientiousness, extraversion, and openness), and facets that make up the five traits [2, 3]. Neuroticism describes the tendency to feel negative emotions, such as sadness, anxiety, and anger, and the intensity of responses to adverse life events [4], and agreeableness the tendency to maintain positive relations with other people, with people high in agreeableness being characterized as warm, caring, selfless, and trusting [2]. Conscientiousness describes the tendency to control impulses and self, be hardworking and strive for achievement, stay orderly, and follow rules [5] and extraversion is the tendency for assertive, energetic, sociable, and spontaneous behavior [6]. Finally, openness is characterized by imagination, curiosity, liberalism, esthetics, and willingness to try new things [7]. All of these traits are heritable and have a neurobiological basis [2, 4–10].

Personality traits are connected to mental health. Especially, neuroticism is associated with mental health, with a high level of neuroticism predicting and preceding psychopathology, particularly mood and anxiety disorders [4, 11–19]. The reason for this connection is unclear, but most evidence points to neuroticism making individuals vulnerable to psychiatric disorders [13]. Other personality traits are also associated with psychopathology [6, 7, 11, 12, 14–19], for example, low agreeableness and conscientiousness correlate with aggression-related disorders and attention deficit hyperactivity disorder (ADHD) [12, 18, 19]. Furthermore, these personality traits are genetically correlated with each other and with mental disorders [8, 20].

Based on decades of studies, it has been proposed that categorical mental health diagnoses would instead form a hierarchical, quantitative construct [21]. This hierarchical construct is called the Hierarchical Taxonomy of Psychopathology (HiTOP) and includes many symptoms and maladaptive traits that make up subfactors. These subfactors, in turn, form six spectra, including, for example, internalizing disorders. Finally, spectra form one large superspectrum, called the p factor. This model would explain many caveats of traditional diagnoses, including strong comorbidity between disorders [8, 22, 23], continuity between normal behaviors and diagnoses [24], and discoveries of more general psychopathology factors [23, 25, 26].

Dogs have been proposed and used as models for human psychiatric disorders [27, 28]. Their symptoms resemble those in humans. For example, both obsessive-compulsive disorder (OCD) and canine compulsive behavior include repetitive behaviors that impair daily functioning [27]. Similarities can also be seen between, for instance, impulsivity/inattention and ADHD [29], aggressive behavior and aggression-related psychopathology, and canine and human separation anxieties [27, 28]. Furthermore, dogs and humans highly resemble each other in social behavior [30]. Dogs are also natural models, as they spontaneously manifest these behaviors [27], unlike induced rodent models that are often very simplified relative to the complex behaviors in humans [31]. Canine models are also genetically [28, 32, 33] and physiologically [32, 34] more similar to humans, and dogs, as our companions, share the same environment with us. Furthermore, loci related to dog behavior, for example, fear, impulsivity, and compulsive behavior, overlap genes related to psychiatric disorders in humans. For example, genomic regions associated with fear and noise sensitivity include neuropsychiatric loci [35–37], DRD4 polymorphisms may be linked to both human ADHD and dog impulsivity [38–40], the same pathways seem to be involved in both human and dog OCD [41, 42], and dog sociability maps to a region harboring human sociability genes [43]. Therefore, the dog seems to be a good model for human behavior.

The association between personality traits and pathological, abnormal, or unwanted behavioral traits has not been studied in dogs. Thus, here, we examined the association of personality with unwanted behavior, namely aggressiveness, fearfulness, and impulsiveness. Many unwanted behavioral traits and psychopathological traits, especially fear and anxiety-related traits, are highly correlated [22, 44–49]. Therefore, we first examined the structure of these unwanted behavioral traits with confirmatory factor analysis (CFA) and then used structural equation modeling (SEM) to include all personality traits, all unwanted behavioral traits, and covariates in the same model. As associations between personality traits and unwanted behaviors are unknown in dogs, our model included paths from all personality traits to all latent unwanted behavioral traits.

## Methods

### Questionnaire

We used our validated [50] dog personality and unwanted behavior questionnaire, which was directed to dog owners. This questionnaire was divided into a background section, a health section, and nine behavior sections: personality, noise sensitivity, fearfulness, aggressiveness, separation-related behavior, fear of surfaces and heights, impulsivity/inattention, cognition, and compulsive behavior, all including several questions related to the dog’s behavior. For a detailed description of the questionnaire, see the Supplementary Material of Salonen et al. [50].

We previously utilized factor analysis to reduce the questionnaire items into factors in each section separately [50], except for the cognition and compulsive behavior sections, which were excluded from this study, as the former section was based on a questionnaire not utilizing factor analysis [51] and the latter was not suitable for factor analysis. This factor analysis reduced the personality questionnaire items into seven personality factors: insecurity, energy, training focus, aggressiveness/dominance, human sociability, dog sociability, and perseverance (Supplementary Table S1). The aggressiveness section was reduced into four components: barking, stranger-directed aggression, owner-directed aggression, and dog-directed aggression (Supplementary Table S1). Impulsivity/inattention section items were based on questionnaire items by Vas et al. [29], and, as in the original study, reduced into two factors: inattention and hyperactivity/impulsivity (Supplementary Table S1). Noise sensitivity, fearfulness, separation-related behavior, and fear of surfaces/heights each comprised one factor (Supplementary Table S1). Factor scores were calculated for each dog, and we used these factor scores in this study.

### Subjects

We utilized the behavior questionnaire data collected in our previous study [50], which, after exclusion of dogs that were deceased over 3 months before answering, dogs with missing birthdates, and duplicate answers included questionnaire responses of 15,371 dogs. From this sample, we excluded dogs whose owners had not answered the personality questionnaire (2506 dogs) and dogs whose owners had answered only the personality questionnaire (1503 dogs). Finally, we also excluded one dog that was an outlier in 3/7 personality traits and one dog with clearly erroneous responses.

The final dataset included questionnaire responses for 11,360 dogs (Supplementary Table S2) of 316 breeds and breed variants. Due to the small number of responses in many breeds, most breeds were grouped based on genetic relationships [52], historical and current purpose, and known behavioral similarities. After grouping, the data included 19 single breeds, 32 breed groups, and mixed breed dogs (Supplementary Table S2) [50].

### Statistical analyses

Before SEM, we utilized CFA to model the structure of unwanted behavioral traits, as they were expected to correlate highly. We used precalculated factor scores instead of defining the latent variables in the CFA, as dog owners could answer the questionnaire sections separately and many owners only answered one or a few of the sections. Therefore, we performed factor analysis for each section separately and removed individuals and questions with more than 20% missing responses, with the mean imputation used for other missing responses [50]. Despite this approach, the CFA models had missing information for unwanted behavioral traits. For these missing data, we used a maximum likelihood approach.

We defined seven competing unwanted behavioral trait structures (Supplementary Fig. S1) and compared them to each other and to a null model, which only included the variances of the original factors. Before CFA, we split the dataset randomly into two equal parts with the package *caret* [53] and fitted the competing models to both datasets to validate the structure. We performed CFA with the package *lavaan* [54] and compared the structures with likelihood ratio tests using the package *nonnest2* [55]. Most of these competing structures were based on the HiTOP (Supplementary Fig. S1a–f) [21, 25], but we also defined a structure based on previous canine behavior studies (Supplementary Fig. S1g) [44–47, 56–62].

We used SEM with the package *lavaan* [54] to examine the relationship between personality and unwanted behavior factor scores. For this model, we used the personality and unwanted behavioral trait factor scores as well as four covariates from the dataset: dog’s age, sex, breed, and socialization score. For missing data present in the model, we used a maximum likelihood approach in SEM, with an option that does not delete cases with missing values in exogenous variables.

Dog’s age was calculated by subtracting its birthdate from the time of questionnaire section response and averaged over all questionnaire sections. Dog’s breed proved challenging to use as a covariate, as *lavaan* cannot handle nominal categorical variables with more than two levels. Coding the dog’s breed as a set of dummy variables was not possible since due to the number of variables (52 breeds and breed groups formed 51 dummy variables) the model failed to converge. Therefore, we calculated the mean trait score for all 52 breed groups in all personality and unwanted behavior factors and range-standardized them between 0 and 1. We used these standardized mean scores as continuous covariates in the model. For example, noise sensitivity score was explained by breed mean scores in noise sensitivity, and human sociability score was likewise explained by breed mean scores in human sociability.

We included socialization as a covariate, as it previously had a highly significant association with fear-related behaviors [59, 63]. Socialization score was obtained by conducting a principal component analysis (PCA) for socialization questions in the background section [50]. This section included seven questions about the dog’s socialization between 7 weeks and 4 months of age. We asked how often the dog met unfamiliar men, unfamiliar women, unfamiliar children, unfamiliar dogs, visited city center, traveled by car, and traveled by public transportation. The response options were never, rarely (1–4 times during puppyhood), sometimes (twice a month-twice a week), often (twice a week-once a day), and very often (several times a day). Before PCA, we used the Kaiser–Meyer–Olkin test for sampling adequacy from the package *psych* [64] to ensure that the data are suitable for the analysis. We used a polychoric correlation matrix and requested the PCA with no rotation with the package *psych* [64]. The best number of components to extract was evaluated with the scree test and Velicer’s minimum average partial test, both of which suggested one component, with all socialization items loading onto the component (Supplementary Table S3). We then extracted the component score for individuals with the estimation method “Thurstone” and used this component score as the socialization score, with a higher socialization score indicating more socialization experiences in puppyhood.

The SEM model was complex and included many variables. Firstly, we defined latent unwanted behavior variables based on the best CFA model. Secondly, we defined regressions for these latent traits, in which each latent trait was explained with all personality traits. Thirdly, as covariates may influence personality and unwanted behavior, we defined regressions for all personality and unwanted behavior traits, in which these traits were explained with the dog’s age, sex, breed, and socialization score. We defined these regressions for each unwanted behavioral trait instead for latent traits, as our previous studies indicated that sex, age, breed, and socialization experiences are differentially associated with correlated unwanted behaviors [44, 59, 63, 65]. Thirdly, we allowed latent traits to correlate freely and defined in total 12 correlations between personality traits based on previous studies (Supplementary Table S10) [17, 20, 66–69]. As many continuous variables were skewed, we used a robust maximum likelihood estimation method. Model fit was evaluated by the comparative fit index (CFI), the Tucker–Lewis index (TLI), the root mean square error of approximation (RMSEA), and the standardized root mean square residual (SRMR).

### Ethics statement

The study was approved by the University of Helsinki Viikki Campus Research Ethics Committee (February 11, 2019). Informed consent was obtained from all participants.

## Results

### Descriptive statistics

We examined the association of personality traits with unwanted behavioral traits in a sample of 11,360 dogs in 52 breeds and breed groups. In total, 52.6% of the dogs were female and 47.4% male. Age varied between 0.18 and 17.48 years, with a mean of 5.21 years (SD = 3.43). The most prevalent breeds and breed groups were Finnish Lapponian dog (*N* = 475, 4.2%), retrievers and flushing dogs (*N* = 458, 4.0%), and Border Collie (*N* = 450, 4.0%; Supplementary Table S2). More descriptive statistics and proportions of missing values are presented in Supplementary Tables S2 and S4.

### CFA models

The dog behavior model provided the best model fit both based on common model fit indices (Supplementary Table S5) and likelihood ratio (Supplementary Tables S6 and S7). This model included four latent variables: fear-related behavior, fear-aggression, aggression, and impulsivity/inattention (Supplementary Fig. S1g and Supplementary Table S8).

### Model fit and covariates

Based on the absolute fit indices, the SEM model achieved good model fit: RMSEA = 0.041 and SRMR = 0.027. However, comparative fit indices indicated only decent model fit: CFI = 0.908 and TLI = 0.874. The covariates age, sex, socialization score, and breed mean score were associated with most unwanted behavioral and personality traits (Supplementary Table S9). Breed mean score was associated with all traits (Supplementary Table S9).

Older age was associated with higher noise sensitivity, barking, stranger-directed aggression, dog-directed aggression, fear of surfaces/heights, inattention, aggressiveness/dominance, and training focus scores and with lower fearfulness, owner-directed aggression, separation-related behavior, insecurity, perseverance, energy, human sociability, and dog sociability scores (Supplementary Table S9).

Being female was associated with higher fearfulness, insecurity, training focus, and human sociability scores and with lower owner-directed aggression, stranger-directed aggression, fear of surfaces/heights, separation-related behavior, inattention, hyperactivity/impulsivity, aggressiveness/dominance, energy, and dog sociability scores (Supplementary Table S9).

Higher socialization score (more socialization experiences in puppyhood) was associated with lower fearfulness, barking, owner-directed aggression, stranger-directed aggression, insecurity, aggressiveness/dominance, and energy scores but with higher fear of surfaces/heights, perseverance, training focus, human sociability, and dog sociability scores (Supplementary Table S9).

### Covariances

**Fig. 1:**
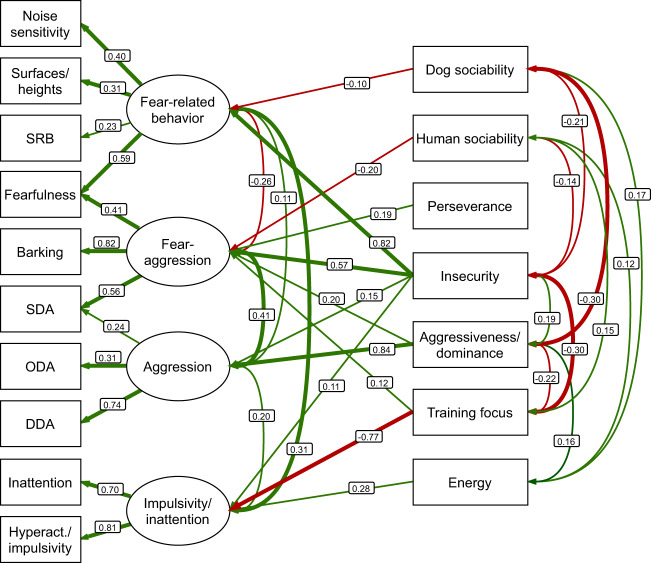
Results of the structural equation modeling (SEM) analysis. Standardized estimates over 0.10 and under –0.10 are included, with paths over 0.30 and under –0.30 in boldface. Positive paths are in green and negative are in red. Covariates (age, sex, breed mean score, and socialization score) are omitted for clarity. All associations are found in Supplementary Tables S8–S11. Surfaces/heights = fear of surfaces/heights, SRB separation-related behavior, SDA stranger-directed aggression, ODA owner-directed aggression, DDA dog-directed aggression, Hyperact./impulsivity hyperactivity/impulsivity.

We defined in total 12 correlations between the seven personality traits, all of which were significant (Supplementary Table S10 and Fig. 1). Of the 6 correlations between latent variables, 5 reached significance (Supplementary Table S10 and Fig. 1). The highest standardized estimates were between fear-aggression and aggression, fear-related behavior and impulsivity/inattention, aggressiveness/dominance and dog sociability (negative), and insecurity and training focus (negative). Covariances with standardized estimates over 0.10 and under –0.10 are shown in Fig. 1.

### Regressions: personality traits associated with unwanted behavioral traits

All personality traits were associated with two or more latent unwanted behaviors (Supplementary Table S9 and Fig. 1). Insecurity and dog sociability scores were associated with all unwanted behavioral traits. The highest standardized estimates were for aggressiveness/dominance score explaining aggression, insecurity score explaining fear-related behavior, and training focus score explaining impulsivity/inattention (negative).

When examining estimates over 0.10 and under –0.10, the most associated explanatory personality trait was insecurity, which was associated with all four latent unwanted behaviors. Of these associations, two were over 0.30. In contrast, energy score was associated only with impulsivity/inattention, perseverance and human sociability were associated only with fear-aggression, and dog sociability achieved a moderate estimate only in fear-related behavior. Regressions with standardized estimates over 0.10 and under –0.10 are shown in Fig. 1. Intercepts and variances are shown in Supplementary Table S11.

## Discussion

We utilized our extensive dog personality and unwanted behavior questionnaire to examine the structure of unwanted behavioral traits with CFA and the association of personality traits with unwanted behavioral traits with SEM. We discovered that many traits were related to each other. Some of these associations were replicated from previous studies, including the comorbidities between unwanted behavioral traits. We also discovered novel findings, for example, the associations between training focus and unwanted behaviors and the correlation between training focus and insecurity. Furthermore, several of these associations, including the strong association of insecurity with fear-related behaviors, were similar to associations in humans, strengthening the use of dogs as models for human behavior and psychopathology.

Based on the content of the dog personality traits [50] and previous literature on human personality, dog personality traits seem to resemble human personality traits. The Insecurity trait was very similar to the human neuroticism trait, with a high score in both indicating negative emotions such as anxiety and worry [3, 4, 70]. Similarly, training focus paralleled the human conscientiousness trait; both were characterized by, for example, self-control and evenness [3, 5, 70]. The aggressiveness/dominance trait seemed to be the opposite of the agreeableness trait [2, 3, 70], and human and dog sociability traits encompassed both agreeableness and extraversion [2, 3, 6, 70]. The energy trait appeared to capture a portion of the extraversion trait, as extraverted individuals are also more active and energetic [3, 6, 70]. Perseverance did not directly resemble any human personality trait, but it seemed to indicate defiant behavior toward the owner, possibly thus capturing a fraction of agreeableness [2, 3].

We examined the structure of unwanted behavioral traits by comparing latent models based on previous literature with CFA. The best fitting model was based on previous dog behavior studies [44–47, 56–62]. This model included four latent traits: fear-related behavior (noise sensitivity, separation-related behavior, fear of surfaces/heights, and fearfulness), fear-aggression (fearfulness, barking, and stranger-directed aggression), aggression (owner-directed, dog-directed, and stranger-directed aggression) and impulsivity/inattention (inattention and hyperactivity/impulsivity). These latent unwanted behavioral traits correlated with each other. The strongest correlations were observed between fear-aggression and aggression and between impulsivity/inattention and both fear-related behavior and aggression. In our previous studies, fearfulness and aggression have also been associated with impulsivity and inattention [44, 65]. Furthermore, the correlation between fear-aggression and aggression is expected, as the aggression trait includes dog-directed aggression, which likely partially also includes aggressive responses evoked by fear. Some studies have also discovered a strong correlation between aggression toward strangers and the owner [58, 71].

Personality traits also correlated with each other, as suspected based on previous studies. The strongest correlations were observed between insecurity and training focus and between aggressiveness/dominance and dog sociability. Both correlations were negative. Aggressiveness/dominance describes aggressive reactions toward other dogs, and therefore, the negative correlation between these traits is not surprising. Similarly in humans, extraversion and agreeableness, both including social behaviors, correlate positively phenotypically and genetically [17, 20, 66, 67]. The negative correlation between insecurity and training focus is more interesting. Training focus was highly negatively associated with impulsivity/inattention, and our previous study showed an association between fearfulness and these ADHD-like traits [65]. Insecure dogs may have difficulties focusing on training, as they likely continuously monitor their surroundings. Conscientiousness and neuroticism are also negatively correlated in humans, supporting our results [17, 20, 66, 67]. Furthermore, conscientiousness and agreeableness are negatively correlated in humans [17, 20, 66, 67], and we discovered a moderate negative correlation between aggressiveness/dominance and training focus. Smaller correlations were present between insecurity and aggression/sociability, between energy and aggression/sociability, and between training focus and human sociability, which also resembled previous results in both dogs and humans [17, 20, 66–69].

Many personality traits were significant predictors of unwanted behavioral traits. Training focus was highly negatively associated with impulsivity/inattention. This is not surprising, as training focus describes the ability to stay focused and orient to tasks, whereas impulsivity/inattention describes the opposite. Paralleling this result, in humans, low impulsivity and conscientiousness are also highly related [19], with impulsivity sometimes regarded as a facet of conscientiousness [5]. We also discovered strong associations between insecurity and unwanted behavioral traits, especially fear-related behavior and fear-aggression. Aggressive behavior is commonly motivated by fear [47, 56, 57], explaining this association between Insecurity and aggression. In humans, neuroticism is the strongest predictor of psychopathology, especially anxiety disorders [4, 11–19], and it is also genetically correlated with psychiatric disorders [8, 10, 20], paralleling our results. Aggressiveness/dominance was strongly associated with aggression. This result was hardly surprising, as the aggressiveness/dominance trait involves aggressive reactions, mostly toward other dogs. However, this result also paralleled psychiatric disorders, as low agreeableness is associated with aggression-related disorders [12, 18]. Finally, energy was positively associated with impulsivity/inattention. This association was also not surprising, as impulsivity/inattention included excessive activity, and dogs very high in energy may be considered excessively active. The association between extraversion and ADHD is less clear and did not show up in a meta-analysis [19], but high extraversion has been proposed to relate to hyperactive/impulsive symptoms of ADHD [20, 72]. Furthermore, high activity level in children is also associated with hyperactive/impulsive symptoms [73]. Perseverance explained some variation in fear-aggression, indicating that it could be related to agreeableness.

Covariates influenced all personality traits and unwanted behavioral traits. Not surprisingly, breed mean score significantly explained variation in personality and unwanted behaviors, indicating that breed indeed influences a dog’s behavior, as discovered in many earlier studies as well [44, 47, 56, 59, 63, 65, 74–78]. Puppyhood socialization also influenced behavior, with more socialized dogs being less insecure but more sociable and trainable. Previous studies have also described this association between puppyhood socialization and adult behavior [59, 63, 79]. Fear of noises, aggressiveness/dominance, and training focus correlated positively with age, while energy level, general fearfulness, and sociability correlated negatively with age, as reported previously [44, 46, 56, 59, 63, 65, 68, 80–82]. Similarly in humans, extraversion decreases and conscientiousness increases with age [5, 83], and the prevalence of anxiety disorders and ADHD decreases with age as well [84, 85]. Female dogs were more fearful, and focused, whereas male dogs were more aggressive, energetic, dog sociable, and showed more separation-related behavior. Similarly, women tend to score higher on neuroticism than men [83]. Furthermore, anxiety disorders are more prevalent in women [84, 86] and ADHD and aggression-related psychopathology in men [84, 87, 88].

Our study has limitations. Firstly, our study was cross-sectional, and thus, causal relationships between personality traits and unwanted behavior cannot be inferred. Secondly, the study utilized an online behavioral questionnaire and collected a convenience sample, which may not represent the entire population and can, thus, influence the results. Thirdly, our study had missing answers in many variables, and we had to use mean imputation to conduct the factor analyses. Fourthly, some unmeasured confounding variables might have influenced the results. In future, longitudinal studies should be designed to examine whether personality traits can predict unwanted behavior in dogs.

In conclusion, this study discovered several associations between personality and unwanted behavioral traits. Many of these associations paralleled associations between human personality and psychopathology. For example, Insecurity, resembling the personality trait neuroticism, was highly associated with unwanted behavioral traits. Similarly in humans, neuroticism is the strongest predictor of psychopathology, especially anxiety and mood disorders. These similarities between dogs and humans suggest that shared genetic and neurobiological factors might underlie these behavioral traits in both dogs and humans. Furthermore, our results indicate that the dog is a good model for both psychiatric disorders and human personality.

## Supplementary information

Supplementary information

Supplementary material: R code

## Data Availability

The pseudonymized data are available as Supplementary Material in Salonen et al. [50].

## Code availability

The R environment is freely available, as well as the packages utilized in the analyses. The R script is available as Supplementary Material.
