# Supplementary material for: Personality traits associate with behavioral problems in pet dogs
Source: Transl Psychiatry. 2022 Feb 23;12:78. doi: 10.1038/s41398-022-01841-0 (PMC8866408; doi:10.1038/s41398-022-01841-0)
Supplement: Supplementary file 1 — Supplementary information [file 41398_2022_1841_MOESM1_ESM.pdf]

Personality traits associate with behavioral problems in pet dogs

Milla Salonen, PhD[1,2,3*], Salla Mikkola, MSc[1,2,3], Emma Hakanen, MSc[1,2,3], Sini Sulkama, MSc[1,2,3], Jenni Puurunen, PhD[1,2,3], Hannes Lohi, PhD, prof.[1,2,3]

[1]Department of Veterinary Biosciences, University of Helsinki, Helsinki, Finland
[2]Department of Medical and Clinical Genetics, University of Helsinki, Helsinki, Finland
[3]Folkhälsan Research Center, Helsinki, Finland

* Corresponding author:
Milla Salonen, postdoctoral researcher
Biomedicum Helsinki
P.O.Box 63 (Haartmaninkatu 8),
FI-00014 University of Helsinki, Finland
Phone: +358 2941 25602
Email: milla.ahola@helsinki.fi

Supplementary Table S1. Personality and unwanted behavior factors and their content.

Section	Trait	Description
Personality	Insecurity	Describes the dog's insecure and fearful tendencies. Highest loading items: insecure, anxious, bold (negative), confident (negative).
	Training focus	Describes the dog's tendency to be focused, obedient, and calm. Highest loading items: focused, distractible (negative), obedient, patient.
	Energy	Describes the dog's tendency to be energetic and playful. Highest loading items: energetic, lazy (negative), slow (negative), active.
	Aggressiveness/ dominance	Describes the dog's tendency to show aggressive and dominant behaviors toward other dogs. Highest loading items: aggressive to dogs of the same gender, dominant, aggressive to dogs of the opposite gender, submissive (negative).
	Human sociability	Describes the dog's tendency to social behavior with people. Highest loading items: sociable with people, affectionate with people, human oriented (prefers the company of people more than the company of other dogs).
	Dog sociability	Describes the dog's tendency to social behavior with other dogs. Highest loading items: indifferent (negative), playful with dogs, sociable with dogs of opposite gender, human oriented (negative).
	Perseverance	Describes the dog's tendency to show persevering and stubborn behavior. Highest loading items: persevering, decisive, stubborn.
Noise sensitivity	Noise sensitivity	Describes the dog's fearful reactions toward fireworks, thunder, gunshot, and other noises. Highest loading items: trembles when hears thunder, trembles when hears fireworks, tail low or between legs when hears fireworks.
Fearfulness	Fearfulness	Describes the dog's fearful reactions towards unfamiliar people and dogs and in a new place or situation. Highest loading items: withdraws when a stranger approaches, moves away if the stranger tries to touch, is suspicious of strangers.
Aggression	Barking	Describes the dog's vocal reactions to unfamiliar people. Highest loading items: barks when a stranger tries to touch (at home), barks when a stranger comes in, barks when a stranger tries to touch (when leashed)
	Stranger directed aggression	Describes the dog's aggressive reactions towards unfamiliar people. Highest loading items: tries to snap/bite when a stranger tries to touch (when leashed), tries to snap/bite when a stranger tries to touch (at home).
	Owner directed aggression	Describes the dog's aggressive reactions towards the owner, specifically when handled or when the owner approaches a resource. Highest loading items: tries to snap/bite when the owners tries to take away bone/food/toy, growls when the owners tries to take away bone/food/toy.
	Dog directed aggression	Describes the dog's aggressive reactions towards other dogs, both when meeting unfamiliar dogs and when familiar dogs approach a resource. Highest loading items: growls when meets unfamiliar dogs, tries to attack when meets unfamiliar dogs.
Fear of surfaces/ heights	Fear of surfaces/ heights	Describes the dog's difficulties to walk on different surfaces and in high places. Highest loading items: difficulties walking on shiny floors, difficulties walking on a slippery floor.

Separation-related behavior	Separation-related behavior	Describes the dog's reactions to being left alone (without people) and its behavior when people are leaving. Highest loading items: pants when owner is leaving, pants when home alone, salivates when owner is leaving.
Impulsivity/ inattention	Inattention	Describes the dog's inability to concentrate. Highest loading items: difficulty to concentrate on a task/play, easy to attract the dog's attention but it loses interest soon, difficulties learning as the dog is careless or other things attract its attention.
	Hyperactivity/ impulsivity	Describes the dog's impulsive reactions and excessive activity. Highest loading items: dog cannot be quiet or easily calmed, dog fidgets all the time, dog is excessive and difficult to control.

Supplementary Table S2. Descriptive statistics of categorical variables. Categorical variables did not have missing data.

Category	Variable	Level	N	%
Covariate	Sex	Male	5380	47.4
		Female	5980	52.6
	Breed group	Australian Shepherd	273	2.4
		Belgian shepherd dogs	193	1.7
		Bernese Mountain Dogs	151	1.3
		Bichon type dogs	204	1.8
		Border Collie	450	4.0
		Brachycephalic dogs	173	1.5
		Bull type terriers	169	1.5
		Chinese Crested Dog	126	1.1
		Collie Rough	181	1.6
		Collie Smooth	142	1.2
		Dachshunds	158	1.4
		English herders	200	1.8
		European sighthounds	188	1.7
		Fighting dogs	153	1.3
		Finnish Lapponian Dog	475	4.2
		German Shepherd Dog	423	3.7
		German spitz related breeds	325	2.9
		Golden Retriever	189	1.7
		Hunting terriers	336	3.0
		Jack Russell Terrier	161	1.4
		Asian primitive dogs	218	1.9
		Labrador Retriever	373	3.3
		Lagotto Romagnolo	190	1.7
		Lapponian Herder	178	1.6
		Livestock guardian dogs	95	0.8
		Mastiff type dogs	276	2.4
		Middle European herders	238	2.1
		Middle European utility dogs	385	3.4
		Miniature Pinscher	131	1.2
		Miniature Schnauzer	164	1.4
		Mixed breed	379	3.3
		Northern companion spitz	178	1.6
		Northern hunting spitz	139	1.2
		Nova Scotia Duck Tolling Retriever	123	1.1
		Other breed	94	0.8

Other companion dogs	277	2.4
Parson type terriers	150	1.3
Pinschers Schnauzers	145	1.3
Pointers	250	2.2
Poodles	233	2.1
Primitive sighthounds	302	2.7
Retrievers & flushing dogs	458	4.0
Scenthounds	94	0.8
Schapendoes	104	0.9
Shetland Sheepdog	279	2.5
Sled dogs	137	1.2
Spanish Water Dog	231	2.0
Teacup dogs	129	1.1
Welsh Corgis	127	1.1
Whippet	175	1.5
White Swiss Shepherd Dog	150	1.3
Yard terriers	288	2.5

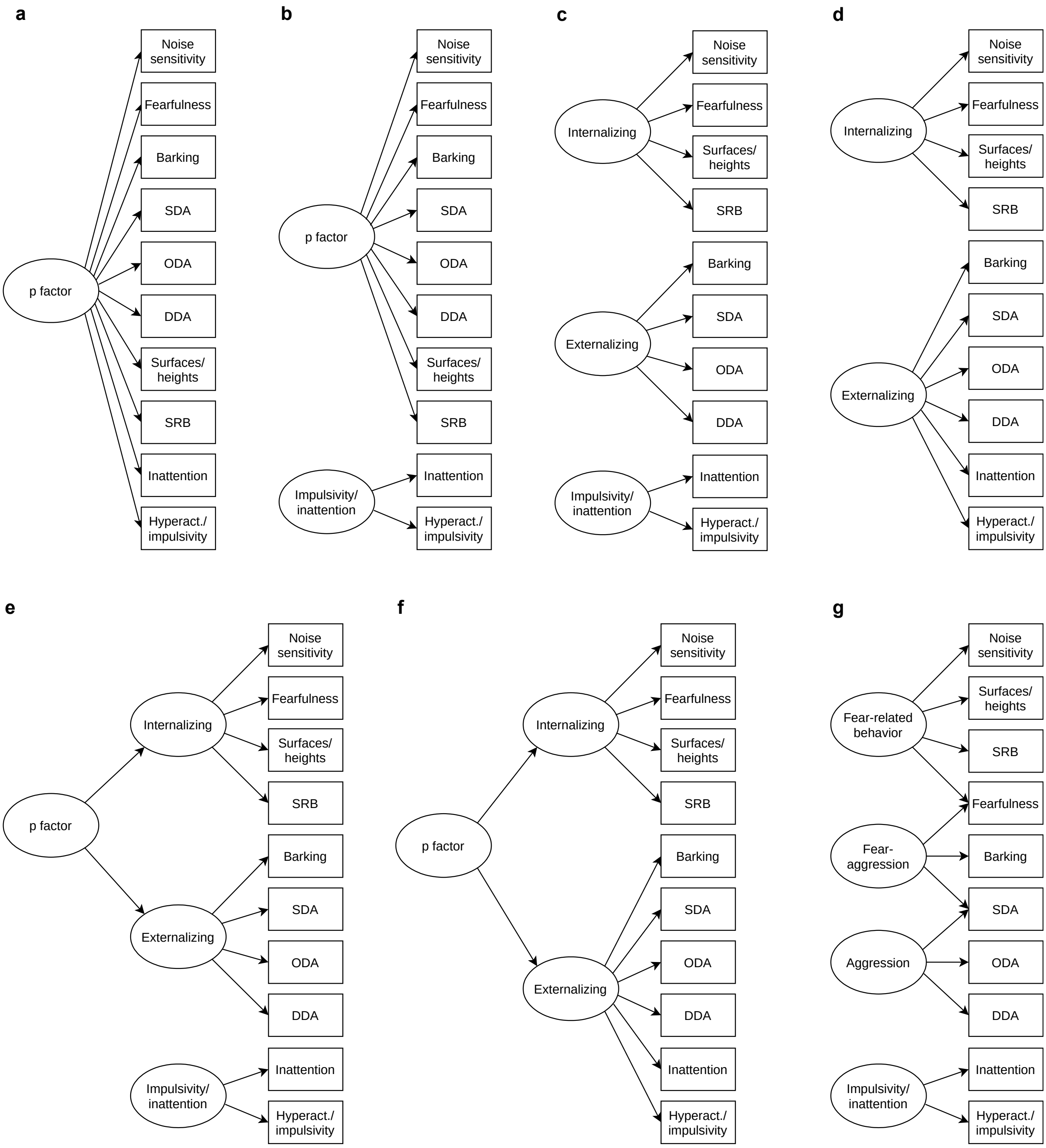

Supplementary Figure S1. Competing CFA models based on previous literature. These models were compared with each other and to a null model, which only included the variances of the observed variables. a) p factor model; b) p factor model and impulsivity/inattention; c) internalizing, externalizing, and impulsivity/inattention; d) internalizing ad externalizing behavior, e) hierarchical model and impulsivity/inattention; f) hierarchical model; g) dog behavior model. Surfaces/heights = fear of surfaces/heights; SRB = separation-related behavior; SDA = stranger directed aggression; ODA = owner directed aggression, DDA = dog directed aggression; Hyperact./impulsivity = hyperactivity/impulsivity.

Supplementary Table S3. Socialization item loadings.

Item	Loading
Met unfamiliar men	0.83
Met unfamiliar women	0.85
Met unfamiliar children	0.46
Met unfamiliar dogs	0.71
Visited city center	0.64
Travelled by car	0.39
Travelled by public transportation	0.44

Cronbach's alpha = 0.71, Guttman's lambda 6 = 0.72.

Supplementary Table S4. Descriptive statistics of continuous variables.

Category	Variable	Range	Mean	SD	% missing
Covariate	Age	0.18 - 17.48	5.21	3.43	0.00
	Socialization score	-3.94 - 2.91	0.00	1.00	17.7
Personality trait	Insecurity score	-1.65 - 4.04	-0.01	1.00	0.00
	Training focus score	-4.87 - 2.48	0.01	1.01	0.00
	Energy score	-3.94 - 2.17	-0.01	1.00	0.00
	Aggressiveness/dominance score	-1.59 - 4.00	0.00	1.00	0.00
	Human sociability score	-4.91 - 1.83	-0.01	1.00	0.00
	Dog sociability score	-3.18 - 2.28	0.00	0.99	0.00
	Perseverance score	-4.16 - 3.8	-0.01	1.00	0.00
Unwanted behavior trait	Noise sensitivity score	-1.36 - 8.75	-0.03	1.16	12.1
	Fearfulness score	-1.51 - 4.84	-0.02	1.07	6.5
	Barking score	-1.39 - 4.73	0.00	0.99	7.3
	Stranger directed aggression score	-1.41 - 8.06	-0.01	0.96	7.3
	Owner directed aggression score	-0.96 - 10.29	-0.02	0.97	7.3
	Dog directed aggression score	-2.29 - 5.10	-0.01	0.99	7.3
	Fear of surfaces/heights score	-0.69 - 6.20	0.00	1.09	18.6
	Separation-related behavior score	-0.63 - 13.23	-0.01	1.12	13.2
	Inattention score	-1.81 - 4.44	-0.01	0.99	10.3
	Hyperactivity/impulsivity score	-1.65 - 4.88	-0.01	0.99	10.3

Supplementary Table S5. Model fit of competing CFA models. CFI = comparative fit index; TLI = Tucker-Lewis index; AIC = Akaike information criterion; BIC = Bayesian information criterion; RMSEA = root mean square error of approximation; SRMR = standardized root mean square residual.

Tested CFA model	Split-sample dataset	CFI	TLI	AIC	BIC	RMSEA	SRMR
Null model	Dataset 1	0	0	147333	147402	0.194	0.191
	Dataset 2	0	0	148911	148981	0.204	0.207
p factor model	Dataset 1	0.622	0.514	141351	141455	0.135	0.091
	Dataset 2	0.666	0.571	141832	141936	0.134	0.087
p factor model + impulsivity/inattention	Dataset 1	0.836	0.782	139299	139407	0.091	0.064
	Dataset 2	0.842	0.791	139960	140068	0.093	0.063
	Dataset 1	0.870	0.817	138972	139087	0.083	0.056

Internalizing, externalizing & impulsivity/inattention	Dataset 2	0.881	0.833	139543	139657	0.083	0.055
Internalizing & externalizing	Dataset 1	0.651	0.538	141074	141182	0.132	0.089
	Dataset 2	0.700	0.603	141470	141577	0.129	0.085
Hierarchical model & impulsivity/inattention	Dataset 1	0.870	0.817	138972	139087	0.083	0.056
	Dataset 2	0.881	0.833	139543	139657	0.083	0.055
Hierarchical model	Dataset 1	0.651	0.524	141076	141187	0.134	0.089
	Dataset 2	0.700	0.591	141472	141583	0.131	0.085
Dog behavior model	Dataset 1	0.955	0.925	138159	138290	0.053	0.033
	Dataset 2	0.955	0.925	138763	138895	0.056	0.034

Supplementary Table S6. Comparison of models in dataset 1. Grey cell: column model obtained better fit. Italicized text: row model obtained better fit. LR: Likelihood ratio.

	**Dog behavior model**	**Hierarchical model**	**Hierarchical model & impulsivity/ inattention**	**Internalizig & externalizig**	**Internalizig, externalizing & impulsivity/ inattention**	**p factor model & impulsivity/ inattention**	**p factor model**
**Null model**	LR = 9209.88, p = 1.66e-08	LR = 6280.37, p < 2e-16	LR = 8386.39, p = 3.67e-09	LR = 6280.37, p < 2e-16	LR = 8386.39, p = 3.67e-09	LR = 8055.45, p < 2e-16	LR = 6001.72, p = 3.01e-09
**p factor model**	LR = 3208.16, p < 2e-16	LR = 278.65, p = 1.03e-08	LR = 2384.67, p < 2e-16	LR = 278.65, p = 1.03e-08	LR = 2384.67, p < 2e-16	LR = 2053.73, p = 8.19e-09	
**p factor model & impulsivity/inattention**	LR = 1154.43, p < 2e-16	*LR = 1775.08, p = 2.2e-08*	LR = 330.94, p < 2e-16	*LR = 1775.08, p = 2.2e-08*	LR = 330.94, p < 2e-16		
**Internalizing, externalizinng & impulsivity/inattention**	LR = 823.49, p = 2.75e-08	*LR = 2106.02, p < 2e-16*	indistinguishable	*LR = 2106.02, p < 2e-16*			
**Internalizing & externalizing**	LR = 2929.51, p < 2e-16	indistinguishable	LR = 2106.02, p < 2e-16				
**Hierarchical model & impulsivity/inattention**	LR = 823.49, p = 2.75e-08	*LR = 2106.02, p < 2e-16*					
**Hierarchical model**	LR = 2929.51, p < 2e-16						

Supplementary Table S7. Comparison of models in dataset 2. Grey cell: column model obtained better fit. Italicized text: row model obtained better fit. LR: Likelihood ratio.

	**Dog behavior model**	**Hierarchical model**	**Hierarchical model & impulsivity/ inattention**	**Internalizing & externalizing**	**Internalizing, externalizing & impulsivity/ inattention**	**p factor model & impulsivity/ inattention**	**p factor model**
**Null model**	LR = 10183.93, p = 2.65e-09	LR = 7463.25, p < 2e-16	LR = 9394.39, p = 5.98e-10	LR = 7463.25, p < 2e-16	LR = 9394.39, p = 5.98e-10	LR = 8973.17, p = 1.52e-08	LR = 7099.55, p < 2e-16
**p factor model**	LR = 3084.38, p < 2e-16	LR = 363.70, p < 2e-16	LR = 2294.84, p < 2e-16	LR = 363.70, p < 2e-16	LR = 2294.84, p < 2e-16	LR = 1873.63, p = 2.65e-08	
**p factor model & impulsivity/inattention**	LR = 1210.76, p < 2e-16	*LR = 1509.92, p = 3.66e-09*	LR = 421.22, p = 1.3e-08	*LR = 1509.92, p = 3.66e-09*	LR = 421.22, p = 1.3e-08		
**Internalizing, externalizing & impulsivity/inattention**	LR = 789.54, p = 8.96e-09	*LR = 1931.14, p < 2e-16*	indistinguishable	*LR = 1931.14, p < 2e-16*			
**Internalizing & externalizing**	LR = 2720.68, p = 1.33e-08	indistinguishable	LR = 1931.14, p < 2e-16				
**Hierarchical model & impulsivity/inattention**	LR = 789.54, p = 8.96e-09	*LR = 1931.14, p < 2e-16*					
**Hierarchical model**	LR = 2720.68, p = 1.33e-08						

Supplementary Table S8. Unstandardized and standardized estimates, Z scores, P values and confidence limits (CL) for the latent variables of the SEM model. All P-values are corrected for false discovery rate. Significant (<0.05) P-values are in bold. CL = confidence limit.

**Latent variable**	**Observed variable**	**Unstand-ardized estimate**	**Stand-ardized estimate**	**Z-score**	**P-value**	**Lower 95 % CL**	**Upper 95% CL**
Fear-aggression	Fearfulness score	1.000	0.409	21.01	**<0.001**	0.371	0.447
	Barking score	1.841	0.816	81.51	**<0.001**	0.796	0.835
	Stranger directed aggression score	1.222	0.561	40.49	**<0.001**	0.534	0.588
Fear-related behavior	Noise sensitivity score	1.000	0.399	32.83	**<0.001**	0.375	0.422
	Separation-related behavior score	0.542	0.225	15.20	**<0.001**	0.196	0.254
	Fear of surfaces/heights score	0.733	0.310	23.35	**<0.001**	0.284	0.336
	Fearfulness score	1.375	0.590	47.88	**<0.001**	0.566	0.614
Aggression	Owner directed aggression score	1.000	0.313	22.25	**<0.001**	0.286	0.341
	Dog directed aggression score	2.400	0.740	59.59	**<0.001**	0.716	0.765
	Stranger directed aggression score	0.756	0.239	16.79	**<0.001**	0.211	0.266
Impulsivity/ inattention	Inattention score	1.000	0.700	78.63	**<0.001**	0.682	0.717
	Hyperactivity/impulsivity score	1.143	0.808	101.52	**<0.001**	0.793	0.824

Supplementary Table S9. Unstandardized and standardized estimates, Z scores, P values and confidence limits (CL) for the regressions of the SEM model. All P-values are corrected for false discovery rate. Significant (<0.05) P-values are in bold. CL = confidence limit.

**Outcome variable**	**Explanatory variable**	**Unstand-ardized estimate**	**Stand-ardized estimate**	**Z-score**	**P-value**	**Lower 95% CL**	**Upper 95% CL**
Fear-related behavior	Insecurity score	0.383	0.824	60.23	**<0.001**	0.797	0.851
	Aggressiveness/dominance score	-0.037	-0.080	-5.83	**<0.001**	-0.107	-0.053
	Perseverance score	-0.008	-0.017	-1.46	0.155	-0.041	0.006
	Training focus score	-0.002	-0.004	-0.26	0.810	-0.031	0.024
	Energy score	-0.035	-0.077	-6.77	**<0.001**	-0.099	-0.054
	Human sociability score	0.015	0.032	2.38	**0.019**	0.006	0.058
	Dog sociability score	-0.047	-0.100	-8.06	**<0.001**	-0.125	-0.076
Fear-aggression	Insecurity score	0.250	0.565	49.70	**<0.001**	0.542	0.587
	Aggressiveness/dominance score	0.090	0.203	16.97	**<0.001**	0.180	0.227
	Perseverance score	0.084	0.189	19.43	**<0.001**	0.170	0.208
	Training focus score	0.053	0.121	11.14	**<0.001**	0.100	0.143
	Energy score	0.014	0.032	3.14	**0.002**	0.012	0.051
	Human sociability score	-0.089	-0.203	-17.10	**<0.001**	-0.226	-0.180
	Dog sociability score	0.019	0.042	3.70	**<0.001**	0.020	0.064
Aggression	Insecurity score	0.045	0.149	12.74	**<0.001**	0.126	0.172
	Aggressiveness/dominance score	0.256	0.836	60.10	**<0.001**	0.809	0.864
	Perseverance score	0.001	0.002	0.17	0.869	-0.019	0.022

	Training focus score	-0.009	-0.031	-2.83	**0.006**	-0.053	-0.010
	Energy score	0.001	0.002	0.20	0.853	-0.018	0.022
	Human sociability score	0.002	0.007	0.64	0.540	-0.014	0.028
	Dog sociability score	-0.019	-0.061	-5.41	**<0.001**	-0.083	-0.039
Impulsivity/ inattention	Insecurity score	0.076	0.111	11.82	**<0.001**	0.093	0.130
	Aggressiveness/dominance score	-0.019	-0.028	-2.81	**0.006**	-0.047	-0.008
	Perseverance score	0.032	0.046	5.20	**<0.001**	0.029	0.064
	Training focus score	-0.525	-0.771	-76.71	**<0.001**	-0.791	-0.752
	Energy score	0.194	0.284	22.40	**<0.001**	0.259	0.309
	Human sociability score	0.024	0.036	4.26	**<0.001**	0.019	0.053
	Dog sociability score	-0.026	-0.037	-3.71	**<0.001**	-0.057	-0.018
Noise sensitivity score	Sex	-0.015	-0.007	-0.72	0.490	-0.024	0.011
	Age	0.054	0.160	16.03	**<0.001**	0.140	0.179
	Socialization	0.024	0.020	1.91	0.062	0.000	0.041
	Noise sensitivity breed mean	0.756	0.160	17.18	**<0.001**	0.142	0.178
Fearfulness score	Sex	0.078	0.036	5.76	**<0.001**	0.024	0.049
	Age	-0.013	-0.040	-5.54	**<0.001**	-0.055	-0.026
	Socialization	-0.024	-0.022	-3.13	**0.002**	-0.036	-0.008
	Fearfulness breed mean	0.498	0.108	17.08	**<0.001**	0.095	0.120
Barking score	Sex	0.002	0.001	0.14	0.887	-0.014	0.016
	Age	0.015	0.052	5.88	**<0.001**	0.035	0.070
	Socialization	-0.072	-0.072	-8.64	**<0.001**	-0.088	-0.056
	Barking breed mean	1.181	0.214	30.42	**<0.001**	0.201	0.228
Owner directed aggression score	Sex	-0.061	-0.031	-3.40	**0.001**	-0.049	-0.013
	Age	-0.009	-0.032	-2.95	**0.003**	-0.052	-0.011
	Socialization	-0.035	-0.036	-3.33	**0.001**	-0.058	-0.015
	Owner directed aggression breed mean	0.633	0.172	19.73	**<0.001**	0.155	0.189
Stranger directed aggression score	Sex	-0.055	-0.029	-3.54	**<0.001**	-0.044	-0.013
	Age	0.009	0.033	3.51	**<0.001**	0.014	0.051
	Socialization	-0.020	-0.021	-2.13	**0.037**	-0.040	-0.002
	Stranger directed aggression breed mean	0.454	0.091	11.58	**<0.001**	0.076	0.107
Dog directed aggression score	Sex	-0.018	-0.009	-1.31	0.205	-0.023	0.004
	Age	0.022	0.077	9.11	**<0.001**	0.060	0.093
	Socialization	-0.011	-0.012	-1.47	0.155	-0.027	0.004
	Dog directed aggression breed mean	0.424	0.088	12.69	**<0.001**	0.074	0.101
Fear of surfaces/heights score	Sex	-0.085	-0.039	-3.95	**<0.001**	-0.058	-0.020
	Age	0.031	0.096	9.46	**<0.001**	0.076	0.116
	Socialization	0.049	0.045	3.92	**<0.001**	0.022	0.067
	Fear of surfaces/heights breed mean	1.258	0.187	15.90	**<0.001**	0.164	0.210
Separation-related behavior score	Sex	-0.184	-0.082	-8.62	**<0.001**	-0.101	-0.063
	Age	-0.016	-0.048	-4.68	**<0.001**	-0.069	-0.028

	Socialization	0.022	0.019	1.79	0.081	-0.002	0.041
	Separation-related behavior breed mean	0.937	0.171	17.12	**<0.001**	0.151	0.190
Inattention score	Sex	-0.053	-0.027	-3.47	**0.001**	-0.043	-0.012
	Age	0.013	0.044	5.15	**<0.001**	0.027	0.061
	Socialization	0.007	0.007	0.77	0.460	-0.011	0.026
	Inattention breed mean	0.673	0.155	19.97	**<0.001**	0.140	0.170
Hyperactivity/ impulsivity score	Sex	-0.030	-0.016	-2.20	**0.031**	-0.030	-0.002
	Age	0.003	0.010	1.12	0.276	-0.007	0.026
	Socialization	-0.002	-0.002	-0.28	0.800	-0.018	0.013
	Hyperactivity/impulsivity breed mean	0.517	0.082	11.15	**<0.001**	0.067	0.096
Insecurity score	Sex	0.088	0.044	4.82	**<0.001**	0.026	0.062
	Age	-0.016	-0.056	-6.28	**<0.001**	-0.074	-0.039
	Socialization	-0.148	-0.149	-13.75	**<0.001**	-0.170	-0.127
	Insecurity breed mean	0.870	0.215	25.06	**<0.001**	0.198	0.232
Aggressiveness/ dominance score	Sex	-0.194	-0.097	-10.98	**<0.001**	-0.115	-0.080
	Age	0.049	0.170	19.34	**<0.001**	0.153	0.188
	Socialization	-0.079	-0.079	-7.58	**<0.001**	-0.100	-0.059
	Aggressiveness/dominance breed mean	1.226	0.278	36.13	**<0.001**	0.262	0.293
Perseverance score	Sex	0.014	0.007	0.79	0.450	-0.011	0.025
	Age	-0.017	-0.060	-6.41	**<0.001**	-0.078	-0.041
	Socialization	0.052	0.053	4.97	**<0.001**	0.032	0.074
	Perseverance breed mean	1.120	0.227	25.58	**<0.001**	0.209	0.244
Training focus score	Sex	0.130	0.064	7.08	**<0.001**	0.047	0.082
	Age	0.039	0.133	15.29	**<0.001**	0.116	0.151
	Socialization	0.111	0.111	10.21	**<0.001**	0.089	0.132
	Training focus breed mean	0.883	0.191	22.92	**<0.001**	0.175	0.207
Energy score	Sex	-0.067	-0.033	-4.02	**<0.001**	-0.049	-0.017
	Age	-0.096	-0.329	-41.39	**<0.001**	-0.344	-0.313
	Socialization	-0.037	-0.037	-3.75	**<0.001**	-0.057	-0.018
	Energy breed mean	1.729	0.348	44.89	**<0.001**	0.333	0.363
Human sociability score	Sex	0.055	0.027	3.03	**0.002**	0.010	0.045
	Age	-0.030	-0.103	-11.48	**<0.001**	-0.121	-0.085
	Socialization	0.077	0.076	7.29	**<0.001**	0.056	0.097
	Human sociability breed mean	1.649	0.224	24.03	**<0.001**	0.206	0.242
Dog sociability score	Sex	-0.215	-0.108	-13.66	**<0.001**	-0.123	-0.092
	Age	-0.131	-0.452	-60.62	**<0.001**	-0.467	-0.438
	Socialization	0.058	0.059	6.37	**<0.001**	0.041	0.077
	Dog sociability breed mean	0.904	0.184	25.49	**<0.001**	0.170	0.199

Supplementary Table S10. Standardized estimates, Z scores, P values and confidence limits (CL) for the covariances of the SEM model. All P-values are corrected for false discovery rate. Significant (<0.05) P-values are in bold. CL = confidence limit.

**Variable 1**	**Variable 2**	**Unstand-ardized estimate**	**Stand-ardized estimate**	**Z-score**	**P-value**	**Lower 95% CL**	**Upper 95% CL**
Insecurity score	Training focus score	-0.280	-0.302	-32.30	**<0.001**	-0.320	-0.283
	Aggressiveness/dominance score	0.173	0.194	18.25	**<0.001**	0.173	0.215
	Dog sociability score	-0.168	-0.206	-21.92	**<0.001**	-0.225	-0.188
	Human sociability score	-0.128	-0.138	-12.49	**<0.001**	-0.159	-0.116
Aggressiveness/ dominance score	Training focus score	-0.195	-0.216	-22.17	**<0.001**	-0.235	-0.197
	Energy score	0.126	0.155	16.14	**<0.001**	0.136	0.174
	Dog sociability score	-0.237	-0.301	-33.40	**<0.001**	-0.319	-0.283
	Human sociability score	-0.061	-0.068	-6.66	**<0.001**	-0.088	-0.048
Training focus score	Dog sociability score	0.024	0.030	3.09	**0.002**	0.011	0.049
	Human sociability score	0.144	0.154	15.19	**<0.001**	0.134	0.173
Energy score	Dog sociability score	0.088	0.118	11.90	**<0.001**	0.099	0.138
	Human sociability score	0.142	0.166	16.73	**<0.001**	0.147	0.186
Fear-aggression	Fear-related behavior	-0.021	-0.255	-6.27	**<0.001**	-0.335	-0.175
	Aggression	0.017	0.405	8.80	**<0.001**	0.314	0.495
	Impulsivity/inattention	0.004	0.039	1.95	0.058	0.000	0.079
Fear-related behavior	Aggression	0.004	0.111	2.43	**0.017**	0.021	0.201
	Impulsivity/inattention	0.028	0.309	9.65	**<0.001**	0.246	0.371
Aggression	Impulsivity/inattention	0.009	0.198	5.39	**<0.001**	0.126	0.270

Supplementary Table S11. Standardized estimates, Z scores, P values and confidence limits (CL) for the intercepts and variances of the SEM model. All P-values are corrected for false discovery rate. Significant (<0.05) P-values are in bold. CL = confidence limit.

Intercepts

**Variable**	**Unstandard-ized estimate**	**Standardized estimate**	**Z-score**	**P-value**	**Lower 95% CL**	**Upper 95% CL**
Fearfulness score	-0.282	-0.261	-9.89	<0.001	-0.313	-0.209
Barking score	-0.580	-0.581	-18.00	<0.001	-0.645	-0.518
Stranger directed aggression score	-0.157	-0.163	-4.82	<0.001	-0.229	-0.097
Noise sensitivity score	-0.589	-0.506	-14.42	<0.001	-0.575	-0.437
Separation-related behavior score	0.052	0.047	1.30	0.207	-0.024	0.117
Fear of surfaces/heights score	-0.472	-0.431	-10.25	<0.001	-0.514	-0.349
Owner directed aggression score	-0.140	-0.144	-3.98	<0.001	-0.215	-0.073
Dog directed aggression score	-0.317	-0.322	-10.36	<0.001	-0.382	-0.261
Inattention score	-0.342	-0.350	-10.71	<0.001	-0.414	-0.286
Hyperactivity/impulsivity score	-0.183	-0.189	-5.99	<0.001	-0.251	-0.127
Insecurity score	-0.424	-0.425	-12.60	<0.001	-0.491	-0.359
Aggressiveness/dominance score	-0.536	-0.540	-15.27	<0.001	-0.610	-0.471
Perseverance score	-0.406	-0.408	-11.01	<0.001	-0.480	-0.335

Training focus score	-0.842	-0.837	-23.03	<0.001	-0.909	-0.766
Energy score	-0.573	-0.572	-14.24	<0.001	-0.651	-0.493
Human sociability score	-1.031	-1.023	-18.52	<0.001	-1.132	-0.915
Dog sociability score	0.638	0.641	20.41	<0.001	0.579	0.703
Fear-aggression	0	0			0	0
Fear-related behavior	0	0			0	0
Aggression	0	0			0	0
Impulsivity/inattention	0	0			0	0

Variances

**Variable**	**Unstandard-ized estimate**	**Standardized estimate**	**Z-score**	**P-value**	**Lower 95% CL**	**Upper 95% CL**
Fearfulness score	0.307	0.263	28.38	<0.001	0.245	0.281
Barking score	0.223	0.224	14.49	<0.001	0.194	0.255
Stranger directed aggression score	0.430	0.464	37.11	<0.001	0.440	0.489
Noise sensitivity score	1.049	0.774	76.13	<0.001	0.754	0.793
Separation-related behavior score	1.139	0.910	118.57	<0.001	0.895	0.925
Fear of surfaces/heights score	1.019	0.849	91.36	<0.001	0.831	0.868
Owner directed aggression score	0.808	0.860	94.04	<0.001	0.842	0.878
Dog directed aggression score	0.375	0.387	21.38	<0.001	0.351	0.422
Inattention score	0.455	0.476	41.23	<0.001	0.453	0.499
Hyperactivity/impulsivity score	0.305	0.326	28.55	<0.001	0.304	0.349
Insecurity score	0.922	0.927	188.41	<0.001	0.917	0.937
Aggressiveness/dominance score	0.864	0.878	159.74	<0.001	0.867	0.888
Perseverance score	0.933	0.941	219.56	<0.001	0.933	0.950
Training focus score	0.937	0.928	197.76	<0.001	0.918	0.937
Energy score	0.771	0.768	114.23	<0.001	0.755	0.781
Human sociability score	0.943	0.930	192.81	<0.001	0.920	0.939
Dog sociability score	0.719	0.727	101.93	<0.001	0.713	0.741
Fear-aggression	0.106	0.542	36.20	<0.001	0.512	0.571
Fear-related behavior	0.065	0.300	14.60	<0.001	0.260	0.340
Aggression	0.017	0.180	7.46	<0.001	0.133	0.228
Impulsivity/inattention	0.123	0.264	25.94	<0.001	0.244	0.284
